# The Intersection of Digital Health, Climate Change, and Nursing Informatics

**DOI:** 10.1055/s-0046-1816595

**Published:** 2026-11-03

**Authors:** Hanna von Gerich, Lorraine J. Block, Sang Bin You, Shauna Davies, Erika Lozada-Perezmitre, Brigitte Fong Yeong Woo, Irem Ozbay, Nader Alnomasy, Sena Chae, Jisan Lee, Mifetika Lukitasari, Mikael Helenius, Pankaj Vyas, Laura-Maria Peltonen, Zerina Lokmic-Tomkins

**Affiliations:** 1 University of Turku, Department of Nursing ScienceTurkuFinland; 2 Vancouver Coastal HealthVancouverCanada; 3 University of Pennsylvania School of NursingPhiladelphiaPennsylvaniaUnited States; 4 University of Regina, Faculty of NursingReginaSaskatchewanCanada; 5 Nursing Faculty, Benemerita Universidad Autonoma de PueblaPueblaMexico; 6 Alice Lee Centre for Nursing Studies, Yong Loo Lin School of Medicine, National University of SingaporeSingapore; 7 Institute of Health Sciences, Deparment of Community Health Nursing, Marmara UniversityIstanbulTurkiye; 8 Department of Nursing, Faculty of Health ScienciesIstanbulTurkiye; 9 Medical Surgical Department, College of Nursing, University of HailSaudi Arabia; 10 Frances Payne Bolton School of Nursing, Case Western Reserve UniversityUSA; 11 College of Nursing, The University of IowaIowa CityIAUSA; 12 Department of Nursing, Gangneung-Wonju National UniversityWonjuGangwon StateRepublic of Korea; 13 School of Population Health, University of New South WalesSydneyNSW2052Australia; 14 School of Nursing, Faculty of Health Science, Brawijaya UniversityMalangIndonesia; 15 Turku University HospitalTurkuFinland; 16 University of Arizona, College of NursingTucsonArizonaUSA; 17 Department of Health and Social Management, University of Eastern FinlandFinland; 18 Wellbeing Services County of North SavoKuopioFinland; 19 School of Nursing and Midwifery, Monash UniversityClaytonVictoriaAustralia

**Keywords:** Climate Change, Nursing Informatics, Digital Health, Climate Resilience, Environment Sustainability

## Abstract

Climate change is a threat to global health. While no one is immune to these effects, their burden falls heaviest on the most vulnerable populations. Nurses are positioned as front-line respondents to these health challenges. Nursing informaticians support the efforts to adapt and mitigate climate change-related health impacts through developing and implementing digital health technologies. This position paper by the International Medical Informatics Association – Student and Emerging Professionals (IMIA-SEP) working group focuses on the importance of nursing informatics and digital health in building more sustainable and resilient strategies to address these global health threats. We present examples of digital health as a tool for climate resilience and discuss the key challenges and considerations. Finally, we present policy recommendations and outline future research areas to support a more sustainable digital future.

## 1. Introduction

Climate change is a threat to global health, exacerbating health disparities [ 1 ]. The World Meteorological Organization has reported that global mean average temperatures of 2024 exceed the record-breaking temperatures of 2023, making 2024 the warmest year and the last decade (2014-2024) the warmest decade on record. These long-lasting variations in the earth’s climate disrupt ecosystems, biodiversity, and health [ 2 ]. The rising incidence, intensity, and duration of extreme weather events, shifting patterns of infectious diseases, and deteriorating air and water quality endanger populations worldwide [ 3 ]. These threats include direct health impacts such as heat illness, and indirect impacts such as pollution-induced and mental health challenges[ 4 ]. By 2050, climate change is projected to lead to 14.5 million additional deaths and increase the risk of infectious disease exposure for 500 million people worldwide [ 5 ].

Nursing informatics (NI) facilitates local and global collaboration and action to improve global health by rapidly deploying efficient strategies [ 6 ]. The adoption of advanced technologies used in care provision has increased the role of NI in supporting the health of people [ 7 , 8 ]. Yet, despite the numerous calls for action posed by the nursing research community, the research and strategies to address the health and environmental risks related to climate change remain inadequately addressed [ 9 , 10 ].

Digital health is the practice of leveraging information and communication technologies to promote health and good care [ 11 ], offering promising nursing informatics (NI)-driven solutions for both climate change mitigation and adaptation [ 12 ]. Strategies to utilize climate-related health data support effective and adaptive responses to environmental health impacts [ 13 ]. Combining digital health solutions with climate change mitigation strategies supports sustainability, promoting resource efficiency and environmental and societal equity [ 14 ]. Mitigative actions include reducing the environmental impacts of healthcare through digital solutions and minimizing the footprint of the digital solutions themselves [ 7 , 13 , 15 , 16 ].

This position paper is motivated by the needs and concerns expressed by the International Medical Informatics Association – Student and Emerging Professionals (IMIA-SEP) working group members to act on the ongoing climate change. NI bridges the gap between nursing science, technology, and data to promote the provision of good care and environmentally sustainable practices. This unique perspective makes NI a critical discipline in addressing healthcare’s contribution to global warming and its associated health impacts. The importance of digital health and NI in climate change response needs to be recognized in order to build more sustainable and resilient strategies to address these global health threats and build climate-resilient healthcare systems.

## 2. Climate Change and Health

Climate change affects global health, sparing no one. Notably, these impacts are not evenly distributed; vulnerable populations such as women, children, the elderly, and low-resource communities bear a disproportionate burden [ 1 , 4 ]. Women often face more significant risks and burdens from the effects of climate change due to social, economic, and cultural factors [ 17 ]. Pregnant women are more susceptible to heat stress and vector-borne diseases, which can lead to complications such as preterm birth and low birth weight [ 18 ]. Children are particularly vulnerable due to their developing bodies and dependence on caregivers [ 19 ]. In disaster scenarios, children are at higher risk of injury, exploitation, and abuse [ 20 ]. For older adult populations, extreme temperatures and poor air quality can exacerbate health issues such as cardiovascular and respiratory diseases [ 21 ]. Physical limitations may hinder evacuation efforts during emergencies, increasing the risk of injury or death [ 22 ]. Poor housing and lack of resilient infrastructure in low-resource communities are more susceptible to floods, storms, and heat waves [ 23 ]. Together with communities vulnerable to land degeneration, indigenous populations, and climate immigrants, these populations are at particular risk of suffering from adverse mental health responses to climate change-induced changes and losses [ 24 ].

Healthcare climate resilience strategies include strengthening policies, financial support, human and material resources, operational processes, and digital health technologies [ 25 ]. NI strives to preserve the delivery of safe care by promoting ethical and sustainable practices, leadership, policy development, education, and research [ 26 ]. Climate change can significantly impair the provision of health services in low- and middle-income countries, leading to a rise in patient admissions whilst decreasing overall healthcare accessibility and the availability of qualified health professionals [ 27 ]. As frontline healthcare providers, nurses are often the first to observe community health changes, enabling prompt responses to climate-induced conditions such as heat stroke or infectious diseases [ 28 ]. Nursing interventions can be successfully used to address the factors increasing the vulnerabilities contributing to adverse health outcomes and health disparities, for example, through knowledge dissemination, community engagement or targeted health campaigns [ 29 ].

Good quality care for vulnerable populations depends on the availability of sufficient tools and processes to support nursing, for example, in gathering relevant health data or supporting patient disease management [ 30 ]. Nurse informaticians have the essential domain knowledge and skills to support the application of digital health technologies in all nursing settings, promoting their responsible development, implementation, use, and education [ 31 ]. Through local and global actions, NI has an important role in transforming data into information, knowledge and wisdom through data governance, management and analytics  [ 26 ]. The pursuit to promote efficient and safe care is carried through all stages of the nursing process, spanning from data generation, access, and use to promoting person-centred, interprofessional care and communication [ 32 ].

## 3. Digital Health as a Tool for Climate Resilience

Nurses represent the largest healthcare profession globally, with their actions having a significant influence on enhancing environmental sustainability and contributing to mitigating climate change [ 33 ]. Telehealth is a notable example of the successful development and implementation of accessible and eco-friendly nursing and NI-driven practices [ 13 ]. It encompasses many cost-effective technological solutions to provide access to care even for the most remote or disaster-affected communities [ 34 , 35 ]. Furthermore, when developing telehealth services, it is possible to design them so that they can deliver excellent service with minimal carbon footprint [ 36 ].Telehealth has proven to be a resilient solution to improve continuity of care during natural disasters and pandemics, such as COVID-19, by enabling remote consultations when access to healthcare facilities is restricted [ 37 ]. While telehealth-based interventions have been proven to promote self-care and enhance patients’ quality of life, their successful utilisation requires strong nurse involvement [ 38 ]. Its adoption and acceptance in rural communities can be hindered due to unrecognised issues related to limited resources or mistrust towards the used technologies or the health care providers [ 39 ]. The optimal use and functionality of telehealth rely on the in-depth knowledge and understanding of the community’s unique characteristics and health issues obtained by the nurses working within these settings [ 34 ]. NI is a key mediator between nursing, technology, and service provision in telehealth implementation [ 40 ].

The application of artificial intelligence (AI) has revolutionized the efficiency, precision, and speed of healthcare data analysis and utilization, offering diverse digital health strategies to prepare for climate change [ 41 ]. For example, nurse informaticians have developed systems that integrate climate data with health record systems to predict and monitor outbreaks of diseases like dengue fever, enabling timely public health interventions [ 42 ]. Tailored, environmentally sustainable AI models could be used to predict and guide timely interventions for potential health risks associated with climate events, including heat-related illnesses [ 43 ], vector-borne diseases [ 44 ], and respiratory issues exacerbated by pollution or wildfires [ 45 ].

Most AI systems that promote nursing care use secondary data from electronic health records or other healthcare information systems [ 48 ]. NI has a key role in ensuring the quality and safety of how this everyday nursing data is documented, stored, accessed, used, and interpreted [ 32 ]. Recent mapping studies have demonstrated the lack of representation of environmental concepts and disaster hazards in one of the most used clinical standard terminologies, SNOMED CT. The identified gaps include concepts related to economic resources, water, air, and sanitation [ 47 ], heat waves, and drought [ 48 ]. Integrating these climate-sensitive outcomes into clinical terminology systems would ensure uniformity in data representation, strengthen climate-resilient healthcare systems, and enhance disaster preparedness, as demonstrated in the field of disaster nursing [ 47 , 48 ].

## 4. Key Challenges and Considerations

Access to digital health technologies remains uneven globally, with sociodemographic factors strongly influencing technology access and use [ 49 , 50 ]. This digital divide risks deepening climate change-related health inequities [ 50 ]. For instance, AI systems depend on real-world data for development, and these structures often have inherent biases and differences included in raw datasets [ 51 ]. Gendered barriers to access, compounded by factors like race, ethnicity, and socioeconomic status, are often overlooked and under-represented in discussions of technical development [ 52 ]. This further underlines the importance for NI to continue with its efforts to incorporate a wider and more diverse range of concepts addressing different health sustainability aspects to standardized clinical terminologies [ 48 ]. A rich representation of concepts could provide more informed data analysis to support monitoring the evolving situation and to share knowledge to better adapt to and equip healthcare across settings. A considerable amount of the required data is produced by nurses throughout the patient care process, highlighting the need to consider how documentation practices and standards support both nursing and knowledge generation [ 53 ].

Whilst many digital health technologies are perceived to help mitigate and adapt to climate change, it is also important to acknowledge their contribution to environmental impacts. For example, while electronic health records reduce paper use and waste, they can emit more greenhouse gases due to energy-intensive infrastructure, highlighting the need for renewable energy integration and less energy-consuming hardware and software in healthcare systems [ 54 ]. Similarly, telehealth can contribute to decreasing transportation-related greenhouse gas emissions and climate change mitigation by reducing the need for in-person visits [ 55 ], but the resources and infrastructure needed to support telehealth provision are usually not comprehensively considered when evaluating their environmental impact [ 56 ]. AI-driven solutions can enhance patient processes, increase cost-efficiency in healthcare, and improve outcomes, addressing healthcare disparities and enhancing outcomes in resource-limited settings [ 57 ]. However, the development and maintenance of these technologies require significant energy resources, contributing to an enlarged carbon footprint, electronic waste generation, and unsustainable mining businesses [ 41 , 58 ].

These examples highlight the complexity and importance of comprehensively considering the environmental sustainability of digital health technologies throughout their entire development life cycle. A clear need exists for the NI community to bring together interprofessional teams to develop standardized evaluation tools factoring in all aspects of technology development and use, beginning from hardware production and system development through the environmental benefits and detriments of using the technologies, all the way to technology retirement [ 56 ]. This systemic thinking approach should also be incorporated into sustainable digital health development by promoting system usability, data governance practices, system interoperability, integration and scalability, and transparency [ 59 ]. Advocating end-user involvement and empowering nurse professionals to collaborate in technology development is crucial in ensuring their safe and ethical use [ 46 ]. Fostering collaborations with global environmental hazard data platforms such as Earth Now and Ambee [ 60 , 61 ] and regional health data ecosystems, like the European Health Data Space [ 62 ] could greatly improve the range and reliability of available data, ultimately creating a more comprehensive and effective global digital health ecosystem. Furthermore, this collaboration would help reduce the carbon footprint associated with data collection and model development, urging the NI community to proactively seek ownership of these opportunities.

The World Health Organization (WHO) has recognized climate change and health as a key priority in healthcare education [ 63 ]. Incorporating climate change and sustainability into nursing education, research and ethical standards is essential to equip the nursing workforce with the skills needed to address emerging trends and to prepare future graduates for the demands of a rapidly evolving healthcare environment. Moreover, it is vital for NI to continue promoting educational strategies to build and enhance the technological competencies of the nursing workforce across healthcare systems globally [ 46 , 64 ].

## 5. Policy Recommendations

The examples provided in this paper highlight the transformative potential and concerns related to digital health technologies in fostering healthcare resilience and environmental sustainability. Active agency and participation of NI in advocating strategies and digital health solutions is called for to prepare for climate change-related health impacts. NI needs to actively seek and promote leadership roles for nursing informatics professionals to advance sustainable digital health development and innovation, resource allocation, and practices. Stronger collaborations between nursing and health informaticians, environmental scientists, information technology engineers, policymakers, and other stakeholders are called for to build systems that bridge the gap between digital health and environmental sustainability. Increased visibility, involvement, and influence of NI professionals in all levels of decision-making and planning facilitate the adoption of digital health solutions that align with clinical and environmental needs.

WHO promotes the adoption of digital health technologies as a strategy for health systems to adapt to and mitigate climate change [ 65 ]. We recommend incorporating environmentally sustainable digital health strategies in national and international health policies and strategies addressing climate change. Furthermore, local and global digital health strategies should be developed and complemented to include policies to promote sustainable practices and digital health technologies, to create approaches to enhance climate-responsive health monitoring, and to improve preparedness and response tactics for exacerbating climate-related events. Lastly, sustainable digital health development frameworks should be developed to incorporate approaches and strategies to narrow existing and impending health inequities by supporting the adoption and use of digital health with marginalized and vulnerable populations most affected by climate change.

Building and evaluating nursing competencies to utilize digital health and comprehend climate-related health risks effectively is essential for delivering high-quality care in the evolving healthcare landscape. This process involves integrating climate-conscious digital health competencies into education and practice, fostering adaptability to technological advancements, and enhancing understanding of environmental health challenges. The nursing workforce should be equipped with adequate skills and knowledge supporting nurse involvement in data governance and management, technology development, implementation, and evaluation, as well as operational, tactical, and strategic decision-making, steering the adoption and use of environmentally sustainable digital health technologies to mitigate and adapt to climate change.

## 6. Future Directions

The WHO defines digital health as the knowledge and use of digital technologies that seek to improve health [ 63 ]. The intersection of digital health and climate change offers important and layered perspectives for citizens, clinicians, and researchers to explore. It is paradoxical that digital health, aimed at helping us to adapt to and mitigate climate change impacts, has its own environmental costs. However, this awareness should inspire action rather than inertia. Digital health and climate resilience must coexist, with ongoing research and innovation driving meaningful progress.

In response, we, as emerging nursing and clinical informaticians, commit to advancing environmentally sustainable digital health as a resource to adapt to and build resilience towards impending health impacts related to climate change, narrow health inequities, and mitigate the effect of digital health technologies on the environment. Following this, we outline areas for future research that we can follow to support these efforts.

The digital health space is slowly evolving to address health and environmental challenges evoked by climate change through innovative solutions. It is vital to research solutions further to promote health through platforms that facilitate service provision and advanced monitoring, forecasting, assessment, and data analysis. It is vital to comprehensively evaluate the efficacy of these solutions in climate adaptation and mitigation and to learn from them. As a first step, we will continue the development and implementation of standardized methods promoting sustainable and ethical development and ecological impact assessment of digital health tools. The framework development will focus on methods to comprehensively and effectively evaluate the carbon footprint of digital health technologies. Moving forward, the validity, reliability, and feasibility of the framework will be evaluated in cooperation with different stakeholders presenting various healthcare domains.

We must look into the data that is needed to promote the development of valid and reliable tools and foster strategies that guide data security, conformity, and interoperability. We need further efforts to evaluate how current data standards can be used or complemented to meet future needs and promote the inclusion of nursing-relevant terminologies tailored to address climate change-induced effects. We will progress our work on data standards that have begun with evaluating the gaps and availability of concepts related to sustainable global health and disaster hazards in SNOMED CT [ 47 , 48 ]. This includes further outlining the data requirements to capture the full range of environmental impacts connected to health and sustainability. Further, we will investigate how these impacts could be incorporated into the existing terminologies and embedded into nursing documentation.

It is also necessary to research nursing documentation practices and how to satisfy our secondary data needs without disrupting the nursing process. We must explore how technology adoption impacts frontline nurses and ensure practices align with their workflows. We will review the existing technology adoption models and how they incorporate practices mitigating the carbon footprint during and after the implementation of digital health technologies. Learning from this, we will investigate how these models could be complemented to consider the environmental concerns of the users and the sustainability goals of the health organizations, as well as to promote more climate-neutral practices.

We must also evaluate the competencies and information needs of professional nurses and the wider NI community and address these gaps through knowledge creation and educational interventions. To begin this effort, we will complement the existing periodic survey of the IMIA-SEP group investigating NI’s current and future trends. We will incorporate elements exploring how digital health technologies are, will, or should be used to mitigate climate change-related health impacts and how NI could contribute to a more sustainable digital future.

In addition to reaffirming the foundation of sustainable digital health through research, we will continue to be vocal advocates of sustainability. This includes calling for integrating digital health strategies with climate change resilience efforts globally. We will also continue to endorse and defend the essential importance of nursing and NI in developing strategies and practices to improve healthcare outcomes in the face of climate disruptions.
